# Gluten-Free Pasta Enriched with Fish By-Product for Special Dietary Uses: Technological Quality and Sensory Properties

**DOI:** 10.3390/foods10123049

**Published:** 2021-12-08

**Authors:** Andrea Aínsa, Alba Vega, Adrian Honrado, Pedro Marquina, Pedro Roncales, José A. Beltrán Gracia, Juan B. Calanche Morales

**Affiliations:** Instituto Agroalimentario de Aragón -IA2, Universidad de Zaragoza-CITA, Miguel Servet. 177, 50013 Zaragoza, Spain; andreaainsa6@gmail.com (A.A.); vega.glez.alba@gmail.com (A.V.); 742755@unizar.es (A.H.); pmarquin@gmail.com (P.M.); roncales@unizar.es (P.R.); jbeltran@unizar.es (J.A.B.G.)

**Keywords:** texture profile analysis, gluten-free, celiac disease, pasta, fish, allergy

## Abstract

Gluten-free pasta enriched with fish can support a nutritive and suitable option for people with celiac disease that allows achieving the benefits of fish consumption, especially the consumption of Ω-3 fatty acids; however, this requires that the pasta has adequate technological and sensory properties. For this purpose, four optimal formulations, obtained with an iterative process, were analyzed to determine the effect of the different ingredients (yellow corn flour, white corn flour, and rice flour) in gluten-free pasta compared to commercial wheat pasta. An evaluation of the color, texture, and technological properties were conducted, and the pasta was sensorially characterized. The enriched gluten-free pasta required shorter cooking times (≈3 min) and was characterized by lower hardness, springiness, gumminess, chewiness, and fracturability, and had higher values of adhesiveness than wheat pasta. In addition, the incorporation of yellow corn gives gluten-free pasta a similarity in color to commercial pasta, with a value of ∆E between 5.5 and 8.0. Regarding the sensory analysis, gluten-free pasta was characterized by slight fishy aromas and flavors with some aftertaste compared to commercial pasta. Finally, the use of different cereals to obtain gluten-free pasta could be a good and feasible alternative despite the technological and sensory modifications observed.

## 1. Introduction

Pasta, in Spanish legislation, is a term employed to describe those products obtained by desiccation of an unfermented dough made with semolina or flour derived from durum wheat, semi-hard wheat, soft wheat, or a mixture of these, and drinking water [1]. Moreover, this product is highly consumed due to its ease of preparation, great versatility, low cost, and good organoleptic properties.

However, this type of pasta cannot be consumed by a part of the population due to celiac disease, which is defined as an autoimmune and multisystemic enteropathy, caused by gluten and prolamins that affect subjects who are genetically predisposed [2,3]. For this reason, people look for gluten-free food with a similar appearance to conventional products, particularly food options that are organoleptically acceptable, with great nutritional features, and are economically accessible. Cereals that affect celiac disease and, therefore, should be removed from the diet are wheat (*Triticum aestivum*), rye (*Secale cereale*), triticale (*Triticum* spp x *Secale cereale*), barley (*Hordeum vulgare*), kamut (*Triticum turqidum*), spelt (*Triticum spelta*), and varieties of oats (*Avena sativa*) that are not guaranteed to be free from cross-contamination and are not certified as gluten-free. Gluten-free foods can be, on the one hand, generics that do not contain gluten by nature and, on the other hand, nongenerics that are divided into conventional ones: those whose formulation or preparation may contain traces of gluten due to cross-contamination, and specific: products specially formulated for people with celiac disease [4]. Gluten-free pasta products are foods that have been specially manufactured, elaborated, and processed to replace gluten and whose gluten level does not exceed 20 ppm of gluten, relative to the product that reaches the final consumer, as indicated in Reglamento 828/2014 [5].

In addition, most gluten-free products, in general, have shown poorer nutritional quality with a low value of minerals and protein with respect to wheat pasta [6]. Further, the elimination of wheat in gluten-free products entails major modifications in nutritional parameters and sensory quality such as color, flavor, and texture [7]. However, due to the possibility they offer, the use of cereals and pseudo-cereals, which are allowed as ingredients in this type of specific formulation, continues to be promoted, since in most cases, it creates a notable improvement of the nutritional properties. To solve the technological difficulties, hydrocolloids, emulsifiers, dairy derivatives, eggs, or soy protein are used in order to improve the texture [8]. According to other authors, an effective alternative strategy to improve nutritional composition and rheological properties of pasta is adding distinct ingredients to the dough, for example, fish [9,10,11,12,13]. Fish is an excellent source of proteins, lipids rich in unsaturated fatty acids, especially Ω3 such as eicosapentaenoic acid (EPA) and docosahexaenoic acid (DHA), minerals, and vitamins (A, D, B6, and B12) [14]. Regarding the above subject, the gluten-free pasta enriched with Mechanically Deboned Meat (MDM) from seabass (*Dicentrachus labrax*) by-products previously dried and milled, represents a new alternative product that could encourage fish consumption from people who do not eat it frequently, which would also help to prevent cardiovascular diseases due to the enriching with bioactive compounds such as unsaturated fatty acids, especially of Ω-3 type, which can remain stable without reaching a sensory rejection as has been seen in previous studies due to the use of an antioxidant [9,11,15]. On top of that, gluten-free pasta would allow taking advantage of the waste of fish processing (offcutting); at the same time, it could be an opportunity to become more environmentally friendly, and thus, allowing for more sustainable activity [16].

However, the change of original ingredients for different ones in pasta could affect physicochemical, sensory, and technological properties. The quality and characteristics of cooked pasta are determined by different parameters such as optimal cooking time, weight gain, hydration, losses during cooking, and texture measured by instrument and sensory analysis [17]. For that reason, gluten-free pasta production focuses on maintaining quality and sensory parameters similar to conventional durum wheat pasta, which could improve its acceptability by consumers.

This research had a focus on the evaluation of the physical, technological, and sensory properties of gluten-free pasta enriched with bioactive compounds from seabass by-products that were added to offer adapted to the celiac population that could contribute to a healthy diet. The main purpose was to obtain a gluten-free pasta with adequate quality and a similarity to traditional wheat pasta.

## 2. Materials and Methods

### 2.1. Raw Material

Fish used to elaborate gluten-free pasta was an offcutting of seabass (*Dicentrachus labrax*) from the filleting process and were provided by a local fish industry (Scanfisk^®^, Zaragoza, Spain). Bones and skin were removed, and the fillets were dipped in saline solution 8%. Then, they were dried in an oven at 60 °C for 24 h with slow air velocity and pulverized to obtain the concentrates. Regarding the cereal, different flours bought in the supermarket were chosen. Gums (xanthan gum and locust bean gum) (SOC Chef 8, DELITÉ 9, Gilca) were used to improve the texture. To choose the definitive gluten-free pasta enriched with seabass concentrate, different formulations were made following the methodology described in Calanche et al. (2019) and Ainsa et al. (2021) [9,11], optimized and adapted to obtain optimal formulations, which are shown in Table 1. Due to the fact that the moisture in the different flours (≈10%) was not significant, the water content was that established for a common pasta. Different gluten-free pastas were compared with a commercial durum wheat pasta, which is denominated as “Control”, and was provided by a local pasta factory (Pastas Romero^®^, Daroca, Spain); the Control pasta was used to analyze the physical, technological, and sensory parameters. Innovative pasta was manufactured with an experimental extrusion machine (Bottene, Mod. Lillodue 14057CE, Marano Vicentino, Italy) [15].

### 2.2. Physical Properties

#### 2.2.1. Estimation of Optimal Cooking Time

The analysis was performed by the visual method following the AACC method 66–50 [18] and by the instrumental method of the Warner–Bratzler shear test for which a rheometer was used (ANAME Scientific Instrumentation, mod. TA-XT2i, Madrid, Spain). The test was performed with the following parameters: pre-test speed: 2 mm/s; test speed: 2 mm/s; post-test speed: 10 mm/s; cutting distance: 15 mm; force threshold: 10 g. After cooking, the pasta was left to cool on a damp paper to avoid drying until it reached 22 °C. The determination was carried out, making a total of 10 consecutive measurements, with a flat probe, to determine, on the one hand, the firmness expressed in kg, described as the maximum force to cut the sample and, on the other hand, the cutting effort, expressed in kg·s.

#### 2.2.2. Texture Profile Analysis (TPA) of Gluten-Free Pasta

The texture profile analysis was made with a texturometer (ANAME Scientific Instrumentation, mod. TA-XT2i, Spain) with a flat cylindrical aluminum and consisted of the application of two compression cycles with a rest time between both (decompression) of 20 s, which allowed the determination of different texture properties: hardness, adhesiveness, cohesiveness, springiness, gumminess, chewiness, resilience, and fracturability. The following conditions were established: test speed: 2 mm/s; sample deformation: 75%; force threshold: 10 g.

#### 2.2.3. Pasta Color

Color readings were taken from three separate points on the surface of cooked pasta (after the pasta was cooked to optimal cooking time, drained, and allowed to stand for 5 min at room temperature). Color measures were made using a colorimeter (Minolta, CM-2002, Osaka, Japan). For each sample, readings were taken 10 times, and the mean value was reported. The following color parameters were recorded: L* (brightness), a* (redness) and b* (yellowness). The color variation produced by the addition of different ingredients was calculated with the total color difference (Δ*E*), from a commercial durum wheat pasta as a reference, using the following formula:

$$\Delta E=\sqrt{{\left(\Delta L^{*}\right)}^{2}+{\left(\Delta a^{*}\right)}^{2}+{\left(\Delta b^{*}\right)}^{2}}$$

where: Δ*L* = L* Fish pasta– L* Standard pasta; Δ*a* = a Fish pasta– a* Standard pasta; Δ*b* = b* Fish pasta– b* Standard pasta.

### 2.3. Technological Properties

#### 2.3.1. Weight Gain and Hydration

The weight gain and hydration of pasta were determined according to the proceedings described by Cleary and Brennan (2006) [19] with the following modifications: 3 g of pasta was cooked in 180 mL of distilled water during the optimal cooking time estimated for each pasta and was cooled in 100 mL of cold water; then, the pasta was dried with absorbent paper and weighed in an analytical balance. To obtain the percentage of the total weight gain of cooked pasta, the following equation was applied:

(2) $$\mathrm{Weight}\;\mathrm{gain}=\frac{\mathrm{Cooked}\;\mathrm{pasta}\;\mathrm{weight}-\mathrm{Raw}\;\mathrm{pasta}\;\mathrm{weight}}{\mathrm{Raw}\;\mathrm{pasta}\;\mathrm{weight}}\times 100\;$$

Once the cooked pasta was obtained, it was dehydrated in an oven at 105 °C for 24 h until reaching a constant weight. The swelling index during cooking was calculated by the following formula:

(3) $$\mathrm{Swelling}\;\mathrm{index}=\frac{\mathrm{Cooked}\;\mathrm{pasta}\;\mathrm{weight}\;\left(g\right)}{\mathrm{Dried}\;\mathrm{pasta}\;\mathrm{weight}\;\left(g\right)}$$

#### 2.3.2. Cooking Losses

The AACC 66–50 method was carried out with the same characteristics as in the previous section (3 g of pasta in 180 mL of water) in their respective optimal cooking times previously estimated [18]. The water resulting from the cooking was collected in crucibles and allowed to evaporate on a stove at 105 °C until reaching a constant weight (24 h). The dry residue was weighed on an analytical balance and determined as a percentage of the total weight of the pasta before cooking.

#### 2.3.3. Moisture

The samples were weighed on the analytical balance; first, they were milled with a laboratory mortar; they were left to dry in an oven at 105 °C for 24 h; they were cooled to room temperature in a desiccator for 1 h; finally, they were weighed again on an analytical balance.

(4) $$\mathrm{Moisture}\;\left(\%\right)=\frac{\mathrm{Raw}\;\mathrm{pasta}\;\mathrm{weight}-\mathrm{Dried}\;\mathrm{pasta}\;\mathrm{weight}}{\mathrm{Raw}\;\mathrm{pasta}\;\mathrm{weight}}\times 100$$

### 2.4. Sensorial Analysis

#### 2.4.1. Sensory Texture Profile (STP)

Sensory texture profile was established following the procedure described in ISO 11036:2020 [20], with adaptations that were made by a panel of expert sensory assessors [21]. Samples were evaluated by a trained panel containing 10 selected assessors [22] who analyzed the parameters: elasticity (assessed with the hands), hardness (when biting with the incisors), disintegration, graininess, pastiness, and stickiness; in a structured scale from 0 to 5. Where 0 represents the absence of the attribute and 5 its maximum intensity.

#### 2.4.2. Quantitative Descriptive Analysis (QDA)

The panel consisted of 10 selected assessors who analyzed different samples according to QDA methodology purpose by Calanche et al. (2019) and Ainsa (2019) for pasta with fish added [9,11]. Attributes evaluated were characteristic aroma of cooked pasta, fish aroma, other odors, hardness (when biting with the incisors), characteristic flavor of cooked pasta, fish flavor, aftertaste (once the sample disappeared from the mouth), other flavors, characteristic color of pasta (intensity in yellow), and the homogeneity of the color. In the sessions, pasta was prepared by cooking in boiling water (100 °C). The samples were served without any type of accompaniment at a temperature of 60 °C following the recommendations of UNE-ISO 6658:2019 [23].

### 2.5. Statistical Analysis

Data obtained in this research were processed by descriptive and inferential statistics using XLSTAT software, Version 2016 (Addinsoft^©^, Paris, France). A univariate analysis was carried out for each considered variable. We conducted a study of distribution to check the normality of data and to detect outlier’s values. Then, Analysis of Variance (ANOVA) tests were applied with a 95% confidence interval and followed by a post hoc test (Fisher) to establish significant differences for different assayed treatments. Moreover, Principal Component Analysis (PCA) was performed to obtain an overview of results obtained by TPA and STP, which allowed us to understand the relationship among parameters taking into account the kind of developed gluten-free pasta. Furthermore, sensory data collected from a selected assessor’s panel when QDA was carried out were used to obtain specific profiles for pasta developed based on Square Cosines Method (sensory characterization).

## 3. Results

### 3.1. Formulations

As shown in Table 1, the durum wheat semolina was replaced in the gluten-free formulations developed in this study by flours of other cereals such as corn (*Zea mays*)—yellow and white—white rice (*Oryza sativa*), and oat (*Avena sativa*) bran. The key aspect to selecting corn was its versatility of use and color, while rice represented an alternative source of starch and protein. It is well known that a decrease in the protein and an increase in the starch content due to the change of the base cereal in the manufacturing of pasta could affect final products that result in a high glycemic index (GI) [24]. Due to the above, increasing the amount of dietary fiber in the pasta through the inclusion of oat bran could, in most of the formulations, reduce GI because it partially replaces the amount of cereal flours.

### 3.2. Physical Properties

#### 3.2.1. Texture Properties

Table 2 shows the optimal cooking time for gluten-free pasta and the results for TPA; concerning ideal cooking time, it was measured instrumentally, using the Warner–Bratzler cutting test (WB) (with a standard deviation of 0.689), and by visual determination (OCT) with a positive correlation between both methods (r^2^ = 0.999).

The estimation of optimal cooking time was very similar using both methods, even coinciding in the YCR pasta, which presented significant differences (*p* < 0.05) concerning the rest of the pasta that could be due to the lack of oat bran in its formulation. The cooking time in the commercial durum wheat pasta used as a control was 10 min; in contrast, gluten-free pasta enriched with seabass concentrate presented optimal cooking times around 3 min.

Regarding TPA, in general, parameters correlated very well with each other (r² ≥ 0.90) and were significantly (*p* < 0.05) different from the types of pasta studied.

According to the results, the control pasta was harder than gluten-free pasta, highlighting WCRO pasta with the lowest value. In relation to adhesiveness, in general, gluten-free pasta was significantly higher (*p* < 0.05) than the control pasta, although the control pasta was statistically similar to YCRO. Concerning springiness, gluten-free pasta was significantly (*p* < 0.05) less elastic than the durum wheat control. Cohesiveness was similar between YCRO and WCRO as well as between YCR and ROG, while the control was significantly different (*p* < 0.05) to all gluten-free pasta. In addition, the gumminess of the control pasta was significantly (*p* < 0.05) bigger than the rest of the gluten-free pasta. About chewiness, control pasta showed significant differences (*p* < 0.05) from gluten-free pasta (+ or −). Finally, control pasta was greater (*p* < 0.05) at fracturability than the rest of the gluten-free pasta.

#### 3.2.2. Color

The color parameters (CIEL*a*b* coordinates and Δ*E*) of gluten-free and control pasta are shown in Table 3.

As it can be seen, the highest value of ΔE corresponds to WCRO pasta due to the low values of a* and b* parameters and the high value of L* for the rest of the pasta. Further, the brightness (L*) showed a significant increase (*p* < 0.05) in WCRO pasta concerning control pasta, while YCR and ROG did not present differences. On the other hand, the a* value was significantly higher (*p* < 0.05) in YCRO pasta with a positive value, while the other pasta had negative values. Finally, the b* parameter showed a greater significant difference (*p* < 0.05) in YCRO pasta, followed by YCR with respect to control and other gluten-free pasta.

### 3.3. Technological Properties

**Figure 1:**
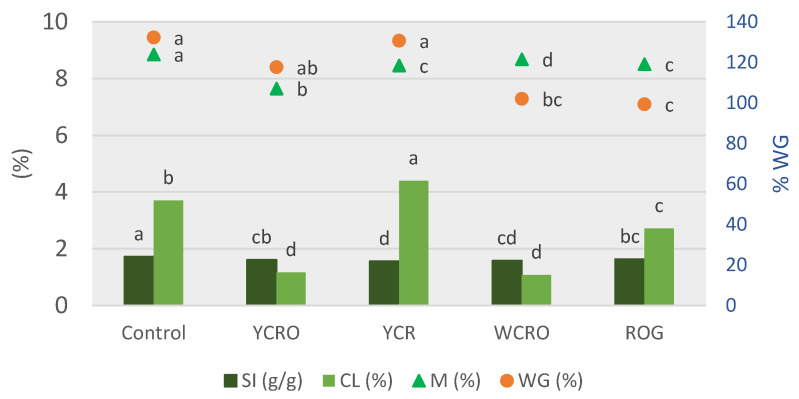
Technological properties of gluten-free and control pasta. Parameters: SI: swelling index, CL: cooking losses, M: moisture, WG: weight gain. Treatment: YCRO: Yellow corn rice oat; YCR: Yellow corn rice; WCRO: White corn rice oat; ROG: Rice oat gums. Different letters represent significant differences (*p* < 0.05) among pasta for each parameter analyzed.

The technological quality parameters of gluten-free pasta enriched with seabass concentrate and control pasta are shown in Figure 1.

The weight gain (WG) is expressed on a scale from 0 to 140%; on the contrary, the moisture (M), the swelling index (SI), and the cooking losses (CL) are expressed on another scale that goes from 0 to 10%. The GW represented in percentage was similar between control pasta, YCRO, and YCR. The SI values showed that gluten-free pasta showed significant differences (*p* < 0.05) concerning control with variations from 1.56 to 1.72%. CL showed less significant differences (*p* < 0.05) in gluten-free pasta than in control pasta, except YCR, which had a larger significant difference. Furthermore, the moisture was around 8.6% for all pasta, with WCRO standing out with the highest value and YCRO with the lowest (*p* < 0.05) value compared to control pasta.

### 3.4. Sensory Parameters

#### 3.4.1. Sensory Texture Profiles (STP)

**Figure 2:**
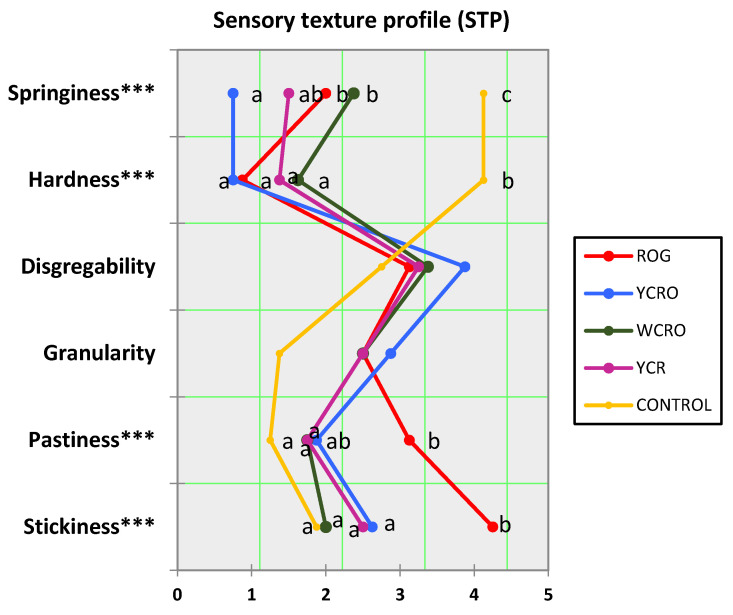
Sensory texture profile (STP) of developed pasta. YCRO: Yellow corn rice oat; YCR: Yellow corn rice; WCRO: White corn rice oat; ROG: Rice oat gums. Different letters represent significant differences (*p* < 0.05) between pasta for each attribute analyzed. *** Parameters with high significant differences (*p* < 0.001) among different pasta.

The sensory texture profile for gluten-free and control pasta evaluated by selected assessors, on a scale from 0 to 5, where 0 is not at all and 5 is a lot, are represented in the semantic differential graph shown in Figure 2. 

The parameters of springiness, hardness, pastiness, and stickiness resulted in significant differences (*p* < 0.05) between pasta. After analyzing the results of the studied texture attributes by the sensory panel and evaluating their performance, it was confirmed that they were capable of correctly discriminating the texture descriptors for each type of pasta. All gluten-free pasta had similar behavior, except for the control durum pasta, which had a different composition concerning the other ones. The sensory analysis confirmed that the most important differences between gluten-free pasta and control were found in hardness and springiness according to TPA. In this way, the highest value of springiness was found in the control pasta, and the lowest value was found in YCRO. Regarding hardness, all gluten-free pasta had similar behavior, although they had significant differences with the control durum pasta. On the other hand, ROG had a higher pastiness and stickiness, showing significant differences with the other types of pasta.

#### 3.4.2. Sensory Profile of Gluten-Free Pasta Developed (QDA)

**Figure 3:**
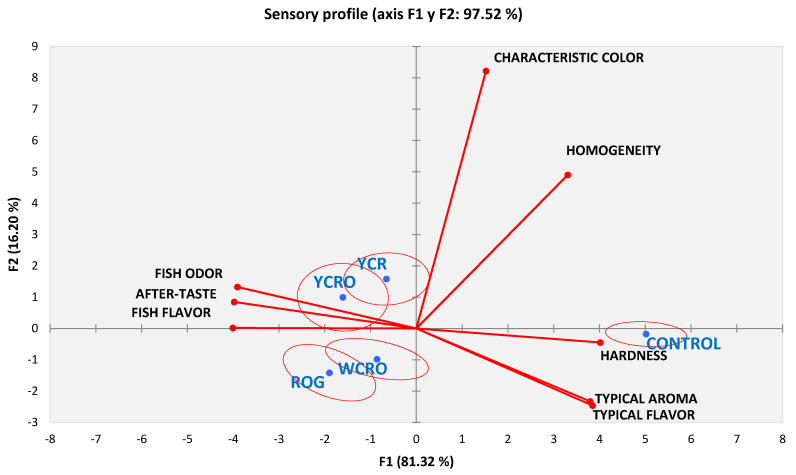
Sensory profile for enriched gluten-free pasta and control pasta. YCRO: Yellow corn rice oat; YCR: Yellow corn rice; WCRO: White corn rice oat; ROG: Rice oat gums.

A quantitative descriptive analysis, which is shown in Figure 3, was performed to characterize the pasta. The performance of the trained assessors’ panel was checked by a panel analysis, not observing significant differences among judges’ evaluations for all studied pasta. The plot from squares cosines study of measurements of trained assessors represented 97.52% of the total variation among kinds of pasta evaluated. The first component described the 81.32% and separated control pasta from gluten-free pasta. Control was characterized by its hardness, typical pasta aroma, typical pasta flavor, homogeneity, and the characteristic color, while gluten-free pasta was associated with fishy flavor and odor and a certain aftertaste. The second component with 16.20% discriminated in the upper part YCRO and YCR while the others (ROG and WCRO) were in the lower part, related to flavor and characteristic aroma of wheat pasta. YCRO was similar to YCR, while WCRO resembled ROG.

### 3.5. Comparative Study between Quality Parameters and TPA

Table 4 shows the linear relationship between the parameters from TPA and technological properties in developed gluten-free pasta. A Pearson correlations analysis was carried out to check associations and significances.

As can be seen, the only parameter of technological properties related significantly (*p* < 0.05) with the TPA parameters was the swelling index. Highlighted the relationship between this index and gumminess and chewiness parameters, all of them related to the viscoelastic behavior of materials.

### 3.6. Comparative Study between TPA and STP

**Figure 4:**
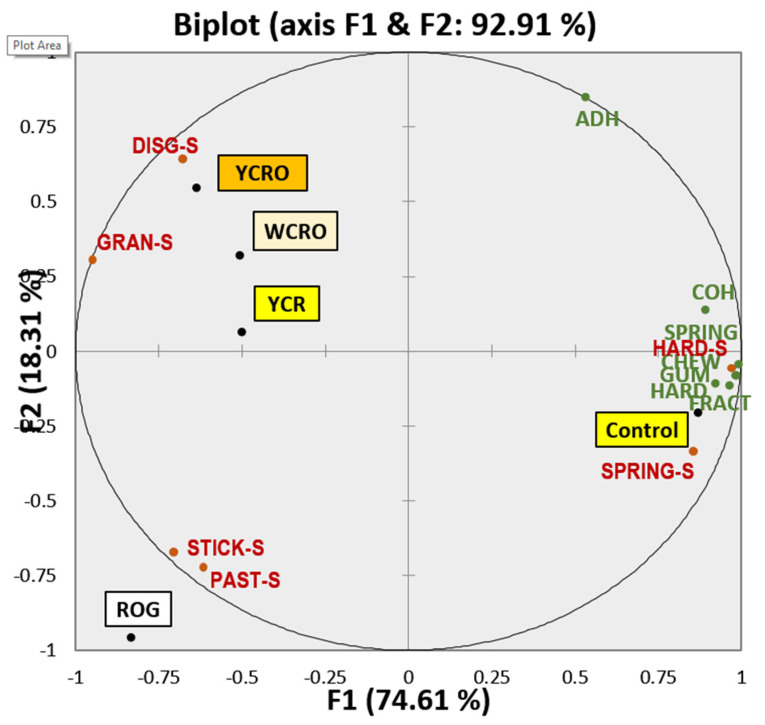
Principal component analysis of texture for enriched gluten-free pasta. HARD: hardness; AHD: adhesiveness; SPRING: springiness; COH: cohesiveness; GUM: gumminess; CHEW: chewiness; FRACT: fracturability; HARD.S: sensorial hardness; DISG.S: sensorial disintegration; GRAN.S: sensorial graininess; SPRING.S: sensorial springiness; PAST.S: sensorial pastiness; STICK.S: sensorial stickiness; YCRO: yellow corn + rice + oat; WCRO: white corn + rice + oat; YCR: yellow corn + rice; ROG: rice + oat + gum.

Due to the great correlations in some cases (r² > 0.881), a principal component analysis (Figure 4) was performed to obtain a global version of the study and observe the relations between TPA and STP for all types of pasta.

The PCA described 92.91% of the variability of the study. The first component was 74.61% and separated control pasta from gluten-free pasta. This allowed finding differences in texture between gluten-free pasta and control pasta. YCRO, YCR, and WCRO were characterized by sensorial disintegration and graininess. On the other hand, ROG was characterized by sensorial pastiness and stickiness, which had a negative correlation with instrumental adhesiveness. Control pasta was associated with the most instrumental parameters (springiness, cohesiveness, fracturability, gumminess, chewiness, hardness, and adhesiveness) and sensorial hardness. The second component, with 18.31%, allowed us to separate treatment by the main ingredient of pasta.

## 4. Discussion

### 4.1. Texture Study of Gluten-Free Pasta Enriched with Fish

As seen in the results, gluten-free pasta had lower cooking times than the control pasta—this could be a consequence of the incorporation of other ingredients. Due to durum wheat was not used in pasta-making, the starch content of the final product and the water required for its gelatinization could be decreased. Further, since it does not contain gluten, the main proteins that form the original structure of traditional pasta decrease when replaced by lower molecular weight and, therefore, require less time to hydrate [25].

Regarding texture profile analysis (TPA), the hardness of pasta is determined by the matrix formed by the gluten network during cooking, in cooperation with other substances such as lipids and starch [26]. The gluten-free pasta did not have this traditional viscoelastic network as it was made from other cereals different from wheat with little or no prolamine in its composition. In addition to the above, the fact that it had in its composition fish concentrate that introduced myofibrillar proteins and fat produced a change of the structure of the dough, causing a weakening in the final product. This modification is because the new ingredients modified the three-dimensional network in the gluten-free pasta, changing the nature and behavior of the material, and as a consequence, modifying the textural properties of the dough, causing mainly hardness [27]. For its part, the adhesiveness increased too due to the weakening of the structure by the change of cereals and the gum addition to improve the consistency. As said before, the new product does not have gluten-forming proteins in its composition. Thus, with the incorporation of fish, rich in myofibrillar proteins and lipids, the observed decrease in springiness due to the absence of wheat proteins is understandable [28]. 

The texture profiles of gluten-free pasta analyzed by instrumental methods showed important changes in their rheological parameters. Highlighted the addition of fish to pasta because it interfered with proper gel formation and, therefore, affected the interaction between starch and cereal proteins, modifying the texture in the final products and increasing the amount of free water [29]. This led to a major change in the rheological behavior of enriched gluten-free pasta developed in this experience.

### 4.2. Color of Developed Pasta

The yellow index (b*) is one of the most important parameters in pasta acceptability due to the characteristic color of pasta [30]. YCRO and YCR pasta, both made with yellow corn, showed higher values of the b* parameter, which could be due to the more intense yellow color provided by this cereal in comparison to durum wheat. All enriched pasta developed in this work showed an appreciable variation with respect to the control, as shown by ∆*E*, due to the characteristic color of cereals used in each pasta-making formulation. Our findings are in agreement with a previous study which showed that pasta with sorghum, suitable for people with celiac disease, had values of ∆*E* with an increase from 20% to 40% [31].

### 4.3. Technological Properties of Developed Pasta

Regarding weight gain, the highest values were found in control wheat pasta and in the yellow corn pasta, which presents a higher amount of amylopectin than the rest of the cereals used. For this reason, these pastas tend to hold more water and swell more [25]. On the other hand, weight gain is reduced by the addition of fish because fish proteins and lipids interact and compete with starch for the absorption of water during cooking, reducing its hydration and gelation [12]. Concerning the swelling index, gluten-free pasta absorbed less water and, therefore, had a decrease in the size of the starch granule responsible for its gelatinization [25,32].

Regarding cooking losses, YCR showed a great difference from the control. This pasta was made without oat bran which differentiates it from the rest. Thus, it could be deduced that oat bran is an ingredient that causes a decrease in cooking losses. Regarding the latter, there is some controversy about the effect of fiber on this technological property. Some studies affirm that enriching pasta with fiber reduces losses since they contribute to the development of the protein matrix by fortifying the structure of pasta [33,34], while others indicate that fiber has greater water absorption capacity and interferes in the formation of the structure and cooking losses will increase [35]. In the same way, the key technological parameter moisture showed that all pasta had values below those established by the BEDCA (9.5%) and Spanish regulation [1,36]. Thus, it could be said that pasta has been made following a correct procedure, in which the applied drying allowed to standardize the moisture content achieve extending pasta gluten-free shelf life.

### 4.4. Sensory Study (Characterization of Product)

According to the sensory texture profile (STP), the principal differences among gluten-free pasta and control pasta were in hardness and springiness; this result coincided withs those provided by the instrumental method (TPA and WB). Pasta made according to ROG formulation was found to be significantly the stickiest and pastiest one. This may be because, in their composition, only rice and oats were used as substitutes for durum wheat. These ingredients modify the rheological properties due to their content of starch and soluble fiber, especially in the case of oats.

As can be seen in the results of the sensory profiles (Figure 3) obtained from QDA, YCRO pasta was similar to YCR treatment, while WCRO was similar to ROG pasta. All gluten-free pasta with fish added made in this study was mainly characterized by a moderate fish odor, a certain aftertaste, and perceivable fish flavor above the typical wheat pasta smell. In the same order of ideas, color and smell in the products were consistent since they coincide with the characteristics that each of the ingredients used in the different formulations is supposed to attribute. At the same time, the control pasta is distinct, as was to be expected, by a typical aroma (cooked pasta) and flavor, with appropriate hardness with characteristic and homogeneous color.

To assess those attributes that must be in an enriched pasta with fish, a comparison was made with the results of a previous study in durum wheat pasta with a fish concentrate where in general, it was characterized by a typical yellow color, farinaceous smell, and typical semolina flavor [37]. Taking all of the above into consideration, the gluten-free pasta enriched with fish that most closely resembles these characteristics is the YCR formulation.

### 4.5. Technological Quality Parameters and TPA

A comparative study demonstrated that even though all quality parameters showed high correlation indices (r^2^), only the swelling index (SI) was statistically significant (*p* < 0.05). This close relationship may have been due to the tendency to increase both aspects, i.e., texture parameters (hardness, gumminess, and chewiness) and technological index. As is known, SI of pasta is an indicator of water absorbed by the starch and proteins during cooking which is utilized for starch gelatinization and protein hydration. Several authors reported that the water absorption capacity depends on the behavior of the proteins denaturation and the function of the amylose/amylopectin ratio, as well as the chain length distribution of amylopectin [38,39]. The above, together with the amylaceous nature of the cereals used to replace wheat semolina, could be the reason that particularly parameters of texture such as hardness, gumminess, and chewiness could have resulted very sensible to amount of water retained in the structural net of the dough.

### 4.6. Global Approach of Texture Study in Gluten-Free Pasta Enriched with Fish

A comparative study between TPA and STP results was carried out, as shown in Figure 4. The principal component analysis takes into account the results for texture from the sensory evaluation and instrumental analysis, considering, at the same time, all kinds of pasta assayed (experimental gluten-free and control pasta) were represented in a unique biplot that collected 92.91% of the total variation of the study. The above fact provided reliability and robustness to the results of the research. In this sense, it is evident how the STP was capable of discriminating of an adequate form both the attributes and the types of pasta analyzed, in contrast with the values obtained for the different determined parameters in TPA, which were located around the control pasta, demonstrating a low discrimination capacity concerning the rest of the treatment with the exception of the adherence located away from the rest of the instrumental parameters of texture. 

It could be claimed that although, in theory, a TPA attempts to mimic what happens in the mouth during the chewing of food [21], it is not as effective in discriminating between traits and attributes as an objective quantitative–descriptive sensory analysis. However, the instrumental determination, as we have already seen, correlates very well with technological parameters, which, together with the information obtained on the organoleptic properties of each type of pasta developed, allows us to have an overall view of the study to have a global understanding and make decisions more accurately.

## 5. Conclusions

The use of cereals other than wheat in enriched pasta with fish could represent a good alternative to contribute to achieving the health of the consumer, especially in the coeliac and/or gluten intolerant population. Gluten-free pasta enriched with fish requires less cooking time than wheat pasta. Further, an effect of oat bran could be seen over the texture properties. The use of different ingredients led to a weakening of the structure of gluten-free pasta and, therefore, to less hardness, springiness, gumminess, chewiness, and fracturability. Concerning color, the addition of yellow corn gave gluten-free pasta a similar color to durum wheat pasta (control). The TPA and STP coincided in pointing out springiness and hardness as the parameters that differentiate the gluten-free pasta from the control pasta. Regarding sensory analysis, all gluten-free pasta were characterized by fish aromas and flavors with a certain aftertaste and a lower hardness in comparison with durum pasta. Finally, according to technological, physical, and sensory parameters, gluten-free pasta made with yellow corn and rice was the most similar to the control pasta.

## Figures and Tables

**Table 1:** Formulations of gluten-free pasta with fish concentrate.

Pasta Formulation	Dry Matter Ingredients (75%)	Wet Matter Ingredients (25%)
CONTROL	Durum wheat semolina	Drinking water
YCRO	Yellow corn flour (45%), rice flour (40%), oat bran (5%), seabass concentrate (10%)	Drinking water + gums (0.6%)
YCR	Yellow corn flour (45%), rice flour (45%), seabass concentrate (10%)	Drinking water + gums (0.6%)
WCRO	White corn flour (45%), rice flour (40%), oat bran (5%), seabass concentrate (10%)	Drinking water + gums (0.6%)
ROG	Rice flour (80%), oat bran (10%), seabass concentrate (10%)	Drinking water + gums (6%)

**Table 2:** Texture properties at the optimal cooking time.

PASTA	WB	OCT (s)	HARD	ADH	SPRING	COH	GUM	CHEW	FRACT
CONTROL	-	-	3726.35 c	−16.01 d	0.77 b	0.67 c	2545.54 c	1993.26 b	758.42 c
YCRO	0.459 a	200	2116.91 b	−25.41 d	0.56 a	0.54 b	1144.35 b	651.48 a	519.52 ab
YCR	2.762 b	210	2208.85 b	−90.93 b	0.58 a	0.43 a	968.00 ab	564.53 a	583.56 b
WCRO	0.408 a	200	1795.78 a	−42.88 c	0.56 a	0.51 b	919.55 ab	517.76 a	461.26 a
ROG	0.396 a	200	1946.21 ab	−257.83 a	0.54 a	0.45 a	896.92 a	488.92 a	496.06 a

Parameters: WB: Warner–Bratzler; OCT: optimal cooking time; HARD: hardness; AHD: adhesiveness; SPRING: springiness; COH: cohesiveness; GUM: gumminess; CHEW: chewiness; FRACT: fracturability. Treatments: YCRO: Yellow corn rice oat; YCR: Yellow corn rice; WCRO: White corn rice oat; ROG: Rice oat gums. Different letters in the same column indicate significant differences (*p* ≤ 0.05) among treatments.

**Table 3:** Color parameters for control and gluten-free pasta.

PASTA	L* (D65)	a* (D65)	b* (D65)	Δ*E*
CONTROL	60.640 bc	−1.608 cd	19.002 c	0.000 d
YCRO	62.369 b	1.074 a	25.769 a	7.788 b
YCR	60.436 c	−1.073 b	21.454 b	5.593 c
WCRO	68.038 a	−2.039 d	8.957 d	12.685 a
ROG	61.408 bc	−1.350 bc	9.893 d	9.332 b

L*: brightness, a*: redness, and b*: yellowness, Δ*E*: color difference. YCRO: Yellow corn rice oat; YCR: Yellow corn rice; WCRO: White corn rice oat; ROG: Rice oat gums. Different letters in the same column indicate significant differences (*p* ≤ 0.05) between pasta for the same coordinate.

**Table 4:** Coefficient correlations (r^2^) between quality parameters and TPA.

TPA	WG (%)	SI (g/g) *	CL (%)	M (%)
HARDNESS	0.714	0.872	0.517	0.406
ADHESIVENESS	0.555	0.119	−0.174	−0.115
SPRINGNESS	0.696	0.840	0.471	0.470
COHESIVENESS	0.433	0.854	0.006	0.233
GUMMINESS	0.624	0.904	0.366	0.388
CHEWINES	0.621	0.901	0.391	0.431
FRACTURABILITY	0.819	0.777	0.662	0.397

WG: weight gain; SI: swelling index; CL: cooking losses; M: moisture. * Statistical significance (*p* < 0.05).

## Data Availability

The datasets generated for this study are available on request to the corresponding author.
